# Heart transplantation in the Netherlands: A national achievement

**DOI:** 10.1007/s12471-018-1090-8

**Published:** 2018-03-08

**Authors:** K. Damman, J. Brügemann, R. A. De Boer, M. E. Erasmus, S. A. J. van den Broek

**Affiliations:** 1 University Medical Center Groningen, Department of Cardiology, University of Groningen, Groningen, The Netherlands; 2 University Medical Center Groningen, Department of Cardiothoracic Surgery, University of Groningen, Groningen, The Netherlands

Dear editor,

We have read with great interest the recent article by Sammani et al. and the accompanying editorial by Y. Pinto in the Netherlands Heart Journal [1, 2]. Evidently, the current level of care for heart transplantation recipients in the Netherlands is high, as presented by the authors. Reasons for improved survival rates are the introduction of better immunosuppressive therapies and the improvement of transplantation care and follow-up. Heart transplant survival has improved over time, despite a change in clinical characteristics such as increased donor age and more frequent use of left ventricular assist devices in recipients. Over the last 30 years the organisation of care for heart transplant patients in the Netherlands has changed as well. In the 1980s, heart transplantation care was limited to two centres, i. e. Utrecht and Rotterdam, but part of the follow-up care (after the actual transplantation in Utrecht) of patients living in the northern and eastern parts of the country was provided at the University Medical Center in Groningen (UMCG) as early as 1991. Indeed, some of the results presented by the authors of the Utrecht programme should thus be regarded as a multicentre and national achievement. The UMCG has been established as an independent heart transplantation centre since 2007 and is responsible for the complete programme, including screening, transplantation and postoperative treatment of these patients in Groningen. The overall expertise in organ transplantation in the UMCG is considerable. In addition to our heart transplantation programme, there are also transplantation programmes for lung, kidney, small bowel, liver, pancreas and bone marrow, as well as multi-organ transplantation programmes, making the UMCG indeed the largest transplantation centre in the Netherlands and the only centre in the country that performs all current types of transplant surgeries.

**Fig. 1:**
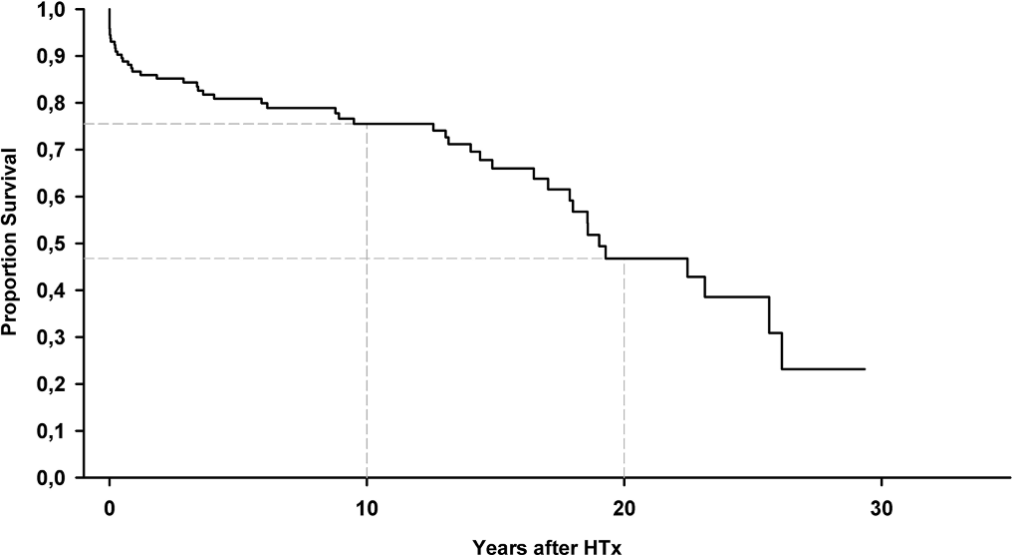
Overall long-term survival after heart transplantation at the UMCG. (*HTx* heart transplantation, *UMCG* University Medical Center Groningen)

As independent heart transplantation centre, the UMCG performed about 10 (7–12) heart transplant procedures per year in the past four years, including 11 procedures in 2017 [3]. In total, the UMCG programme performed over 70 transplants since 2007, including combined heart-lung transplantations, a multi-organ transplantation that is performed exclusively at the UMCG [4]. Similar to the findings from Utrecht, we have been able to reach good survival statistics for the patients having received either a heart transplant or follow-up care at the UMCG (which includes some patients who received transplants in Utrecht after 1991), with a 10-year and 20-year survival rate of 76 and 47%, respectively (Fig. 1). The statistics for heart transplantations at the Erasmus Medical Center in Rotterdam were remarkably similar, as published earlier [5].

In conclusion, the University Medical Center Groningen has been established as an independent heart transplantation centre as of 2007, and it has since demonstrated similar post-transplant survival rates compared with both Utrecht and Rotterdam.
